# Current shreds of evidence on the anticancer role of EGCG in triple negative breast cancer: an update of the current state of knowledge

**DOI:** 10.1186/s13027-020-0270-5

**Published:** 2020-01-10

**Authors:** Sabrina Bimonte, Marco Cascella, Antonio Barbieri, Claudio Arra, Arturo Cuomo

**Affiliations:** 1 Division of Anesthesia and Pain Medicine, Istituto Nazionale Tumori - IRCCS – “Fondazione G. Pascale”, Naples, Italy; 2 S.S.D. Sperimentazione Animale, Istituto Nazionale Tumori - IRCCS – “Fondazione G. Pascale”, Naples, Italy

**Keywords:** Triple-negative breast cancer, (−)-epigallocatechin 3-gallate, Anticancer activity, Apoptosis

## Abstract

Triple-Negative Breast Cancer (TNBC), represents a subtype of breast cancer in which the estrogens receptor (ER) negative, the progesterone receptor (PR) negative and the human epidermal growth factor receptor 2 (HER2) negative, are not expressed. Thusly, TNBC does not respond to hormonal therapies or to those targeting the HER2 protein receptors. To overcome this flawed issue, new alternative therapies based on the use of natural substances, as the (−) - epigallocatechin 3-gallate (EGCG), has been proposed. It is largely documented that EGCG, the principal constituent of green tea, has suppressive effects on different types of cancer, including breast cancer, through the regulation of different signaling pathways. Thus, is reasonable to assume that EGCG could be viewed as a therapeutic option for the prevention and the treatment of TNBC. Here, we summarizing these promising results with the scope of turn a light on the potential roles of EGCG in the treatment of TNBC patients.

## Background

**Fig. 1:**
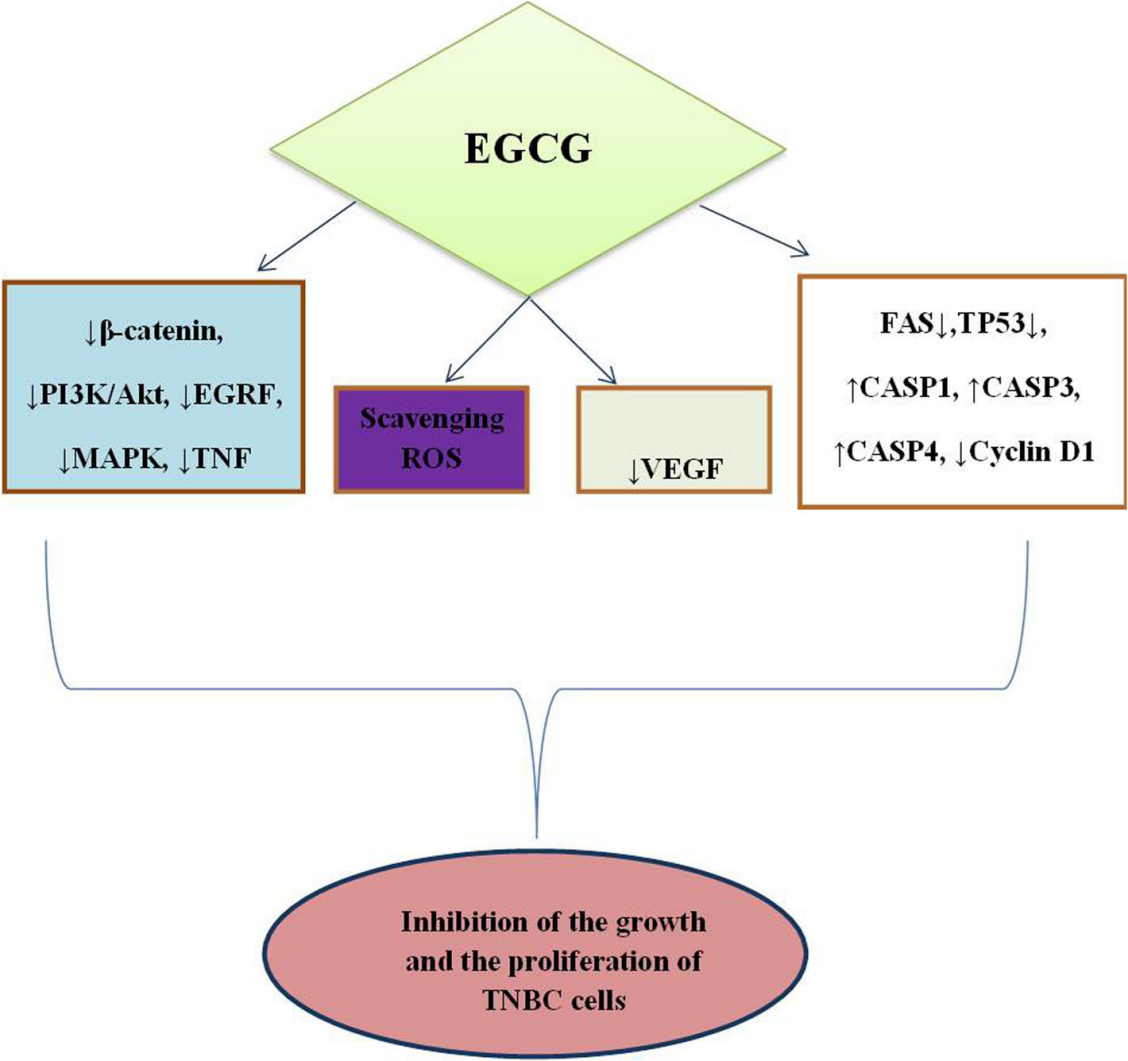
Molecular mechanism underlying the antitumor effect of EGCG in Triple Negative breast cancer cells. EGCG: epigallocatechin-3-gallate; PI3K: phosphatidylinositol-3 kinase; EGRF: epidermal growth factor receptor; MARK: mitogen-activated protein kinase; TNF: tumor necrosis factor; ROS: reactive oxygen species; VEGF: vascular endothelial growth factor; FAS: fas cell surface death receptor; CASP1: caspase 1; CASP3: caspase 23; CASP4: caspase 4; TP53: tumor protein p-53; TNBC: triple negative

Triple-negative breast cancer (TNBC), accounting for 15–20% of breast cancer with diagnosis, is an aggressive disorder with a poor prognosis frequently founded in African women with mutations in BCRA1 gene [1]. This tumor, classified as basal-like cancer on the basis of its morphology [2], does not express the estrogen receptor (ER), the progesterone receptor (PR), and the human epidermal growth factor receptor 2 (HER2) negative (neu) markers [3]. Thus, TNBC fails to respond to therapies targeting the HER2 protein receptors or to conventional treatments. Regarding eziopathogenesis of breast cancer, a recent study highlighted a link between the bovine leukemia virus (BLV) and breast cancer, suggesting that the DNA of BLV in mammary tissue could be considered an important marker for breast cancer [4]. Moreover, Banerjee S et al., identified the microbial signatures associated with TNBC, by using a pan-pathogen array technology in order to provide the new diagnostic potential for this type of cancer viruses [5]. Unfortunately, there are no successful therapies for TNBC, thus new elective treatments engineered on the use of natural substances, have been developed in order to change the conventional schedule of therapies. Particularly, several studies identified the polyphenols as adjuvants to chemotherapeutic drugs in TNBC cells [6, 7]. Many reports highlighted the role of EGCG against the progression of different types of cancer, including breast cancer, thought the modulation of different molecular pathways [8–15]. Specifically, it has been showed that EGCG inhibited breast cancer cell growth by enhancing the chemotherapeutic-induced cellular apoptosis [16], and by modulating different molecular signaling pathways as the nuclear factor-κB (NF- κβ), the epidermal growth factor receptor (EGFR), the mitogen-activated protein kinase (MAPK) and the phosphatidylinositol-3 (PI3) kinase [17–20]. Moreover, it has been proved that EGCG retarded the growth of MDA-MB-231 human breast cancer cells, by inactivating the β-catenin signaling pathway (Fig. 1) [21]. Overall, these findings demonstrate that EGCG could be considered as a new agent for the treatment of patients suffering from TNBC.

## An overview of the role of EGCG in TNBC cells growth: findings from in vitro studies

Accumulated in vitro studies highlighted the role of EGCG in the inhibition of TNBC cells growth, through the regulation of various molecular pathways (Table 1). The first study on this issue was conducted by Braicu et al. on Hs578T cells. Authors showed that the galloylated flavan-3-ols, and particularly EGCG, inhibited the cell proliferation by inducing the apoptosis, due to their ability to scavenge reactive oxygen species (ROS). Moreover, flavan-3-ols showed antioxidants or pro-oxidants properties depending on the dose and the exposure time of the cell culture [22]. Particularly, it was demonstrated that EGCG reduced or increased the concentration of reactive oxygen species (ROS) respect to controls and to others galloylated flavan-3-oils, at doses of 10 and 100 μM. In a subsequent investigation, the same authors demonstrated that EGCG (20 μM) inhibited the proliferation of Hs578T cells after 48 and 72 h of treatments, mainly by activating the apoptotic processes. By using the PCR-array technology, it has been shown that EGCG altered the expression of many genes involved in different molecular pathways (Table 1). EGCG downregulated the expression of several anti-apoptotic genes (insulin-like growth factor-1 receptor: IGF1R; induced myeloid leukemia cell differentiation protein: MCL1) while increased the expression of other genes (B-cell lymphoma 2: Bcl2; associated athanogene 3: BAG3; receptor-interacting serine/threonine-protein kinase 2: RIPK2; X-Linked Inhibitor of Apoptosis: XIAP) probably due to resistance to cancer treatment [23]. Later on, a suppressive role of EGCG on TNBC cell migration was associated with VEGF expression inhibition [24], suggesting that EGCG could be used to arrest breast tumor invasion. In a fascinating study, Braicu et al. proposed that the combination of p53 siRNA and EGCG, through the activation of apoptosis and autophagy, potentiated the antitumor effects of EGCG in Hs578T cells [25].

**Table 1:** In vitro findings on the role of EGCG in TNBC cells

*Cell lines*	*Dose of EGCG*	*Molecular targets*	*Ref.*
Hs578T	10, 100 μM	ROS ↓ (at 10 μM) ROS ↑ (at 100 μM)	[22]
Hs578T	20 μM	CASP1, CASP3, CASP4, PYCARD, BAG3, RIPK2, XIAP↑ IGF1R, MCL1, TNF↓;	[23]
Hs578T	10 μM	VEGF↓	[24]
Hs578T	10 μM plus 40 nmol p53-siRNA	PYCARD, BAD, BAG3, CD40LG, CD40, CD27, TNFRSF1A, TNFSF8, CARD6, LTBR, BAK1↑; IGF1R, MCL1, TNFRSF25, BRAF, FAS, TP53↓;	[25]
MDA-MB-231	25, 50, 75, 100, 200 μM. Pretreatment with 25 μM LY294002 or 5 μM wortmannin + plus 200 μM EGCG added after 24 h	β-catenin, p-AKT, cyclin D1↓	[21]
MDA-MB231	IC_50_ (μM) of Epigallocatechin 3- Gallate Synthetic Analogues: G28, G37, G56, M1, M2, G75 (−)-	FASN↓ PARP ↑	[26]
MCF-7, MDA-MB- 231, MDA-MD-157, HCC1806	3 μM m SAHA, 5 μM EGCG	miR-221/222, p27, ERα, PTEN, E-cadherin, N-cadherin,	[27]

*Abbreviations*: *EGCG* epigallocatechin-3-gallate, *ROS* reactive oxygen species, *CASP1* caspase 1, *CASP3* caspase 3, *CASP4* caspase 4, *PYCARD* apoptosis-associated speck-like protein containing a card, *IGF1R* insulin-like growth factor-1 receptor, *MCL1* induced myeloid leukemia cell differentiation protein, *TNF* tumor necrosis factor, *BAG3* bcl2 associated athanogene 3, *RIPK2* receptor-interacting serine/threonine-protein kinase 2, *XIAP* X-linked inhibitor of apoptosis protein, *VEGF* vascular endothelial growth factor, *BAD* BCL2 associated agonist of cell death, *TNFRSF1A* tumor necrosis factor receptor superfamily member 1A, *TNFSF8* tumor necrosis factor ligand superfamily, member 8, *CARD6* caspase recruitment domain family member 6, *LTBR* lymphotoxin beta receptor, *BAK1* BCL2 antagonist/killer 1, *TNFRSF25* tumor necrosis factor receptor superfamily member 25, *BRAF* b-raf serine/threonine-protein, *FAS* fas cell surface death receptor, *TP53* tumor protein p-53, *p-AKT* phospho-AKT, *PARP* poly(ADP- ribose) polymerase, *SAHA* suberoylanilide hydroxamic acid, *miR-221/222* microRNA 221/222, *ERα* estrogen receptor alpha, *PTEN* phosphatase and tensin homolog

A different molecular mechanism underlying the inhibitory role of EGCG on TNBC cell growth was reported by Hong et al. [21]. Specifically, the authors firstly evaluated a presumable association between β-catenin expression and clinical conditions of female patients with invasive ductal carcinoma, and then they examined the effect of EGCG on β- catenin expression in triple-negative breast cancer cells, MDA-MB-231. Results of western blot analysis conducted in breast cancer and normal tissue of female enrolled in this study, revealed that the expression levels of β-catenin were higher in breast cancer tissue than in normal tissue. Accordingly, EGCG decreased the cell viability of MDA-MB-231 cells in a dose-dependent manner, by downregulating the expression of β-catenin, cyclin D1, and phosphorylated Akt (p-AKT). Moreover, pre-treatment of MDA-MB-231 cells with peptidase inhibitor 3 (PI3) kinase inhibitors, (LY294002 or wortmannin) potentiated the suppressive effect of EGCG, added after 24 h, on β-catenin expression. Taken together, these data suggest that EGCG is able to inhibit the growth of MDA-MB-231 by interfering with the β-catenin signaling pathway.

Encouraging results on the effects of (−)-Epigallocatechin 3-gallate synthetic analogs on TNBC cells were depicted by Crous-Masò. In this study were generated three diesters (G28, G37, G56), two G28 derivatives and two monoesters M1 and M2. Then, these compounds were tested in vitro on MDA-MB-231 cells. Specifically were evaluated their cytotoxic effects, their inhibition of lipogenic enzyme fatty acid synthase (FANS) and their apoptotic activities. Data emerged from these experiments, showed that all compounds blocked the activity of FASN. Particularly, the monoesters inhibited the growth of MDA-MB-231 cells more than the diesters, and only the monoesters induced the apoptosis detected by poly (ADP-ribose) polymerase (PARP) cleavage. Altogether, these results suggest that these polyphenolic compounds, particularly the monoesters, due to their inhibitory role on FASN, could represent a new strategy for TNBC treatment [26].

A recent study evaluated the effects of Suberoylanilide hydroxamic acid (SAHA) histone deacetylase (HDAC) inhibitor and EGCG on the growth and the proliferation of TNBC cells [27]. Results showed that these substances reduced the expression of miR-221/222 and N-cadherin and increased the expression of p27, phosphatase and tensin homolog (PTEN) estrogen receptor alpha (ERα) and E-cadherin. These findings, likewise associated with reduced activity of DNA methyltransferase (DNMTs), indicate that SAHA and EGCG reduced the growth and the proliferation of TNBC cancer cells, probably by acting on epigenetic mechanisms.

## The antitumor effects of EGCG in TNBC mouse models: a current state of knowledge

The antitumor effects of EGCG on TNBC have been also confirmed by in vivo experiments, as summarized in Table 2. A first study was conducted by Thangapazham al [20]. Authors showed that EGCG (administered at the dose of 1 mg/animal in 100 μl of distilled water for 10 weeks), and GTP (green tea polyphenols, 1% for 10 weeks) decreased the proliferation and increased the apoptosis of tumors of TNBC mouse model. Importantly, these results were firstly proved in vitro. Specifically, GTP (10–150 μg/ml) and EGCG (1–200 μg) inhibited cell growth in a dose-dependent manner. Moreover, an arrest of the cell cycle at G1 phase, confirmed by a downregulated expression of Cyclin D, Cyclin E, Cyclin-dependent kinase 4 (CDK 4), Cyclin-dependent kinase 1 (CDK 1) and proliferating cell nuclear antigen (PCNA), was observed in cells treated with GTP and EGCG. An interesting study conducted on the same TNBC mouse model tested the effect of a novel EGCG prodrug on tumor growth [28]. Specifically, this compound, named Pro-EGCG (1), was engineered to enhance the stability of EGCG by introducing the peracetate-protecting groups to the reactive hydroxyls of EGCG. By using this approach, the authors showed that in MDA-MB-231 cells, Pro-EGCG (1) was converted to its parent compound EGCG that was then accumulated. Thus, the cells treated with Pro-EGCG (1) (50 μmol/L), showed increased levels of proteasome inhibition, growth suppression and apoptosis compared to cells treated with EGCG. These data were also confirmed in vivo by injecting the MDA-MB-231 cells in nude mice, followed by treatment with Pro-EGCG (1) (50 mg/kg) or EGCG (50 mg/kg) for 31 days. Data emerged from this experiment showed that Pro-EGCG (1) inhibited breast tumor growth and proteasome, while induced the apoptosis compared to EGCG, suggesting its use for TNBC prevention and treatment. Similar reports were showed by Yang et al. [29] with the utilization of the prodrugs of EGCG’s flour-substituted analogs: Pro-F-EGCG2 (Pro-F2) or Pro-F-EGCG4 (Pro-F4) and Pro-EGCG 1. These compounds (50 mg/kg administered by s.c. injection daily for 31 days), equally, inhibited the growth and the proteasome and enhanced the apoptosis in breast tumors of mice.

**Table 2:** In vivo experiments on the antitumor effects of EGCG on TNBC: an update

Animal models	Drug	Dose	Effects	Reference
Xenograft mouse model (MDA-MB-231 cells injected into the dorsal subcutaneous tissue of mice)	EGCG GTP	1 mg/0.1 ml/mouse of EGCG in drinking water for 10 weeks; 0,1% GTP for 10 weeks;	EGCG and GTP decreased proliferation and increased apoptosis of tumors of TNBC- bearing mice.	[20]
Xenograft mouse model (MDA-MB-231 cells injected into the dorsal subcutaneous tissue of mice)	EGCG Pro- EGCG (1)	50 mg/kg Pro- EGCG(1) for 31 days; 50 mg/kg EGCG(1) for 31 days;	Pro-EGCG (1) targeted the tumor cellular proteasome and inhibited the growth of tumors of TNBC-bearing mice.	[28]
Xenograft mouse model (MDA-MB-231 cells injected into the dorsal subcutaneous tissue of mice) injected into the right axilla of mice)	Pro-F- EGCG2 (Pro-F2) Pro-F- EGCG4 (Pro-F4) Pro- EGCG (1)	50 mg/kg by *s.c.* injection daily for 31 days, Pro-F2; 50 mg/kg by *s.c.* injection daily for 31 days, Pro-F4; 50 mg/kg by *s.c.* injection daily for 31 days, Pro-EGCG (1)	Pro-F2 and Pro-F4 induced proteasome inhibition and apoptosis induction and similar to Pro-EGCG (1), inhibited the growth of tumors of TNBC-bearing mice.	[29]

*Abbreviations*: *EGCG* epigallocatechin-3-gallate, *GTP* green tea polyphenols, *TNBC* triple negative breast cancer, *Pro-EGCG(1)* peracetate-protecting groups to the reactive hydroxyls of (−)-EGCG, *Pro-F2* prodrugs of fluoro-substituted EGCG analog-42, *Pro-F4* prodrugs of fluoro- substituted EGCG analog-4, *s.c.* subcutaneously

## Limitations of the use of EGCG into clinical practice and future perspectives

The aforementioned results strongly indicate that EGCG can restrain the growth and the proliferation of TNBC by acting on different molecular pathways. Thus, EGCG might be viewed as an alternative treatment for TNBC patients. Notwithstanding these encouraging pre-clinical outcomes, no clinical trials have been conducted, until now, on TNBC patients. Lamentably, the data obtained from in vivo and human epidemiological studies on other types of breast cancer, are inconsistent and in some cases contradictory [30]. Moreover, EGCG possesses poor bioavailability and poor stability and these highlights change between in vitro and in vivo conditions [31, 32], thus representing a limitation for EGCG’s use in human populations. To conquer this issue, new strategies based on the use of nanoparticles (NPs) or micro-particles in which EGCG is encapsulated, have been effectively built to improve EGCG’s stability and bioavailability [33–39].

Further investigations will be important not only to improve EGCG oral bioavailability, yet additionally to stabilize this compound in the stomach related tract. Overall, EGCG could be firmly viewed as a powerful inhibitor of TNBC progression.

## Data Availability

Data sharing not applicable to this article as no datasets were generated or analyzed during the current study.

## Abbreviations

BAD: BCL2 associated agonist of cell death
BAG3: bcl2 associated athanogene 3
BAK1: BCL2 antagonist/killer 1
BRAF: b-raf serine/threonine-protein
CARD6: Caspase recruitment domain family member 6
CASP1: Caspase 1
CASP3: Caspase 3
CASP4: Caspase 4
EGCG: Epigallocatechin-3-gallate
ERα: Estrogen receptor alpha
FAS: Fas cell surface death receptor
GTP: Green tea polyphenols
IGF1R: Insulin-like growth factor-1 receptor
LTBR: Lymphotoxin beta receptor
MCL1: Induced myeloid leukemia cell differentiation protein
miR-221/222: MicroRNA 221/222
p-AKT: Phospho-AKT
PARP: Poly(ADP- ribose) polymerase
Pro-EGCG(1): Peracetate-protecting groups to the reactive hydroxyls of (−)-EGCG
Pro-F2: Prodrugs of fluoro-substituted EGCG analog-42
Pro-F4: Prodrugs of fluoro- substituted EGCG analog-4
PTEN: Phosphatase and tensin homolog
PYCARD: Apoptosis-associated speck-like protein containing a card
RIPK2: Receptor-interacting serine/threonine-protein kinase 2
ROS: Reactive oxygen species
SAHA: Suberoylanilide hydroxamic acid
TNBC: Triple-negative breast cancer
TNF: Tumor necrosis factor
TNFRSF1A: Tumor necrosis factor receptor superfamily member 1A
TNFRSF25: Tumor necrosis factor receptor superfamily member 25
TNFSF8: Tumor necrosis factor ligand superfamily, member 8
TP53: Tumor protein p-53
VEGF: Vascular endothelial growth factor
XIAP: X-linked inhibitor of apoptosis protein
